# Endoscopic endonasal approach for resection of pediatric chordoma with brainstem invasion

**DOI:** 10.3171/2019.10.FocusVid.19421

**Published:** 2019-10-01

**Authors:** Kumar Abhinav, David Hong, Carol H. Yan, Peter Hwang, and, Juan C. Fernandez-Miranda

**Affiliations:** 1 Departments of Neurosurgery and; 2 Otolaryngology, Stanford University School of Medicine, Stanford, California

**Keywords:** endoscopic endonasal approach, chordoma, clivus, posterior clinoidectomy, video

## Abstract

A 14-year-old boy had undergone an orbitozygomatic craniotomy for a pontine lesion. There was growth on surveillance imaging with involvement of posterior clinoids, clivus, and left pons suggestive of chordoma (Fernandez-Miranda et al., 2014b). An endoscopic endonasal approach was undertaken involving full upper and midclival exposure including bilateral posterior clinoidectomy (Fernandez-Miranda et al., 2014a; Truong et al., 2019a, 2019b). The internal carotid artery was skeletonized to maximize exposure and facilitate safe resection. The tumor was removed from between the dural layers of the midclivus while preserving the interdural abducens nerve (Barges-Coll et al., 2010). The brainstem component was resected while preserving the pontine perforators. Postoperative diagnosis was chordoma with MRI demonstrating complete resection. The patient was intact postoperatively.

The video can be found here: https://youtu.be/g6SQ5JVK0Ko.

## Transcript

Here we present an endoscopic endonasal approach for resection of a pediatric chordoma in a 14-year-old patient that underwent a previous orbitozygomatic approach for what was thought to be a brainstem lesion. Pathology came back as chordoma and has progressively grown after the initial resection.

### 0:38 Pertinent anatomy

It is key to understand the anatomy of the upper petroclival region for this operation and the described transcavernous posterior clinoidectomy to achieve complete resection of the involved tumor. We need to understand the medial wall of the cavernous sinus anatomy so that we can access the cavernous sinus and mobilize the wall away from the carotid artery by cutting the inferior ligament and the inferior hypophyseal artery. In addition, it is key to understand the anatomy of the pons, the basilar artery, the perforating branches, and the different segments of the abducens nerve.

### 1:16 Surgical approach, posterior clinoidectomy, and tumor resection

So we have performed now our endonasal endoscopic approach. We are working transclival exposing the right carotid artery on the paraclival segment down to the lacerum segment. Now we are exposing the paraclinoidal artery on the left side. Now we are drilling the midclivus and we start seeing tumor invading in between the two layers of dura, as is so typical for these clival chordomas. I am now getting a good margin inferiorly to make sure that is all clean of tumor. Now we are working on removing the posterior clinoids on both sides as well as the dorsum sella. We are Dopplering the anterior wall of the cavernous sinus so that we can directly incise the anterior wall without injuring the carotid artery. Once we open the anterior wall of the cavernous sinus, we obtain significant venous bleeding that is actually easily controlled with gentle packing. As we open the cavernous sinus, we identify the inferior hypophyseal artery. I am now opening the anterior wall of the cavernous sinus superiorly all the way towards the clinoidal segment of the carotid artery. We are now cutting the floor of the cavernous sinus giving access to the posterior clinoid. We see tumor invading the posterior clinoid. This happens to be a very prominent posterior clinoid and a large inferior hypophyseal artery located just above it. I am trying to carefully dissect the posterior clinoid from the dura but the dural attachments are very robust. I am seeing here now the inferior parasellar ligament. This is being transected. This attaches the medial wall of the cavernous sinus to the carotid and after transecting this ligament, I can now mobilize better the posterior clinoid and the medial wall of the cavernous sinus, but still we have the attachment by the inferior hypophyseal artery, which I have to coagulate and transect, and after this I can finally cut the dura that surrounds the posterior clinoid. This is going to allow me to now use a dissector to gently remove the posterior clinoid. We can see its posterolateral extension and I can now remove the posterior clinoid completely, the dorsum sella, and now we are removing the posterior clinoid on the left side of the patient. In this case, since I have more space, I can do it in an extradural fashion, but still I get venous bleeding from the cavernous sinus, which is easily controlled. Finally, removing the dorsum sella, posterior clinoid allows me to remove all the tumor that is invading the interdural space, and this outer layer of dura is going to be extensively removed. I am seeing now the sixth nerve at the interdural segment now on the left side, and now on the right side I am cutting the dura around the Dorello’s canal, making sure with electrical stimulation that I am preserving the abducens nerve. It is very important to reach the limit; this is the inferior petrosal sinus bleeding there and I trim all this dura because it’s potentially involved with tumor. That dural thickening there is the petrosphenoidal or Gruber’s ligament that is located just posterior to the abducens nerve. Here are some anatomy pictures showing this complex anatomy of the abducens nerve, Dorello’s canal, petrosphenoidal ligament, and the different segments of the abducens nerve. I continue dissecting the dura that is thickened and potentially involved with tumor. I am doing the similar operation on the patient’s left side, preserving the sixth nerve but at the same time maximizing the resection of the dura, and that is the penetration of the tumor through the inner dural layer and through the arachnoid into the subarachnoid space. I am doing a wide dural opening because all this dura is also potentially involved and needs to be transected. Unfortunately, because of the previous operation, there are significant adhesions between the dura, the arachnoid, and the basilar artery. This requires very careful meticulous dissection to prevent a vascular injury. We are finally able to remove all this dura and separate it safely from the basilar artery. There is a small remnant of tissue that is very adherent to the wall of the basilar artery. This requires again very careful meticulous technique to achieve a complete resection but at the same time without causing any vascular injury. This is done with sharp dissection, mostly avoiding pulling that could injure the basilar artery. After this portion is finished, we identify the invasion of the tumor into the brainstem. We are going to find a plane to safely dissect this pontine perforating branch. There is also significant scar tissue in this area, which makes the operation more difficult, but I am now finding a plane between brainstem and tumor and I can finally start scooping the tumor out from the inside of the brainstem. I find another pontine perforating branch inferiorly and posteriorly, and this has to be very carefully preserved. After dissecting the vessel and separating it from the tumor, I can continue opening the capsule of the tumor and entering the brainstem so that I can find a plane of dissection between tumor and neural tissue. The tumor is removed in a piecemeal fashion now using the two suctions in a very controlled fashion. I can remove any residual tumor within the pons. In fact, there is a membrane of tissue that I actually removed carefully sent to the pathologist and they came back saying there was no tumor, so we obtained a complete tumor resection.

### 8:35 Reconstruction of the skull base defect

The reconstruction is done with a collagen layer, then fascia lata, then a fat graft, and importantly an extended healthy nasoseptal flap. Postoperatively the tumor was completely removed as evidenced on the MRI. Patient did very well with no complications, normal pituitary function, no CSF leak, and an intact sixth nerve.
